# JSH practical guidelines for hematological malignancies, 2023: II. Lymphoma5. Diffuse large B-cell lymphoma, not otherwise specified (DLBCL, NOS)

**DOI:** 10.1007/s12185-025-03997-z

**Published:** 2025-05-28

**Authors:** Ken Ohmachi

**Affiliations:** Department of Internal Medicine, Division of Hematology and Oncology, Tokai University School of Medicine, 143 Shimokasuya, Isehara-City, Kanagawa 259-1143 Japan

**Keywords:** DLBCL, R-CHOP, Pola-R-CHP

## Overview

Diffuse large B-cell lymphoma, not otherwise specified (DLBCL, NOS) as defined in the 2017 WHO classification accounts for over 30% of all non-Hodgkin lymphomas (NHL) in Japan, making it the most prevalent form of NHL [1, 2]. DLBCL can occur as primary disease or through histologic transformation from other low-grade B-cell lymphoma, and is an inhomogeneous group of various entities. Immunohistochemical staining (using CD10, bcl-6, and IRF4/MUM1) and gene expression profiling are used to classify DLBCL by cell of origin into the germinal center B-cell subtype and activated B-cell/non-germinal center B-cell subtype [3]. However, due to the lack of clear evidence that stratification of treatment based on these subtypes improves prognosis, the practice is generally not recommended [4–6].

This chapter will cover DLBCL, NOS as well as certain DLBCL subtypes (T-cell/histiocyte-rich large B-cell lymphoma; primary cutaneous DLBCL, leg type; and EBV-positive DLBCL, NOS) and related entities (primary mediastinal large B-cell lymphoma, intravascular large B-cell lymphoma [IVLBCL], DLBCL associated with chronic inflammation, lymphomatoid granulomatosis, and ALK-positive large B-cell lymphoma). The subtypes of DLBCL are collectively referred to as DLBCL.

The Ann Arbor classification [7] is widely used for staging, and the International Prognostic Index (IPI) as a prognostic model [8]. Recently, a group of cancer centers in North America proposed the NCCN IPI as a prognostic indicator for DLBCL in the rituximab era [9]. This index has more specific sub-classifications of age and serum LDH, and is considered to enable more accurate assignment of patients to the low- or high-risk group than the IPI. The 2014 Lugano classification [10], which is based on FDG-PET/CT results, is widely used for response assessment.

## Algorithms

### Algorithm 1

**Fig. 1:**
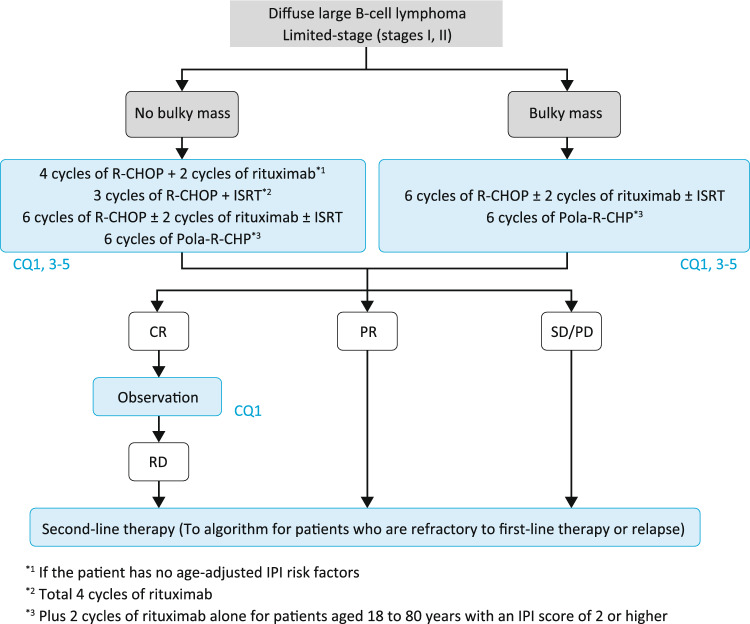
Treatment algorithm for limited-stage DLBCL

In the context of DLBCL treatment, limited-stage disease corresponds to clinical stage I and contiguous stage II in the Ann Arbor classification, because when considering radiotherapy for limited-stage disease, involved-field radiotherapy (IFRT) was considered to be indicated for contiguous stage II disease treatable within a single radiation field (Fig. 1).

A large randomized controlled trial established combined modality treatment (CMT) consisting of 3 cycles of the CHOP regimen (cyclophosphamide, doxorubicin, vincristine, and prednisolone) followed by IFRT as the standard therapy for limited-stage DLBCL [11]. However, CMT with 3 cycles of CHOP plus IFRT is not recommended for patients with a bulky mass. After the introduction of rituximab, recommended treatments have become either CMT with 3 cycles of R-CHOP plus local radiotherapy (formerly IFRT, but currently involved-site radiation therapy [ISRT] is recommended as advances in diagnostic imaging and radiotherapy have enabled appropriate reduction of the target volume and dose) or 6 cycles of R-CHOP alone for limited-stage DLBCL without a bulky mass (maximum tumor diameter > 10 cm or mediastinal mass with a maximum diameter exceeding one-third of the maximum chest diameter), and 6 cycles of R-CHOP for DLBCL with a bulky mass [12, 13]. There are no clear guidelines for when to perform CMT with 3 cycles of R-CHOP plus ISRT versus 6 cycles of R-CHOP without ISRT, and thus treatment should be selected with consideration to whether 6 cycles of chemotherapy would be feasible. If the patient does not have a mass with a maximum diameter exceeding 7.5 cm and has no age-adjusted IPI risk factors, 4 cycles of R-CHOP followed by 2 additional cycles of rituximab alone is another recommended standard therapy (CQ1) [14]. Six cycles of polatuzumab vedotin with R-CHP (pola-R-CHP) (plus two cycles of rituximab alone) is another recommended standard therapy for patients aged 18 to 80 years with an IPI score of 2 or higher, with or without a bulky mass [15]. ISRT should be performed for patients who would not be good candidates for chemotherapy.

Though one controlled trial found that consolidation therapy with IFRT at 30 Gy after 8 cycles of CHOP yielded no improvement in survival [16], a subgroup analysis of that trial and a large retrospective analysis of patients who received radiotherapy did show improvement in survival [17, 18]. These results indicate that post-chemotherapy ISRT (with a radiation field of similar or smaller size than that at initial diagnosis) may be considered to treat the site where the lesion was located before treatment, particularly in the case of a bulky mass.

If a complete response (CR) is obtained after treatment, the treatment approach should be shifted to observation. If the response is a partial response (PR) or worse, salvage chemotherapy should be performed.

### Algorithm 2

**Fig. 2:**
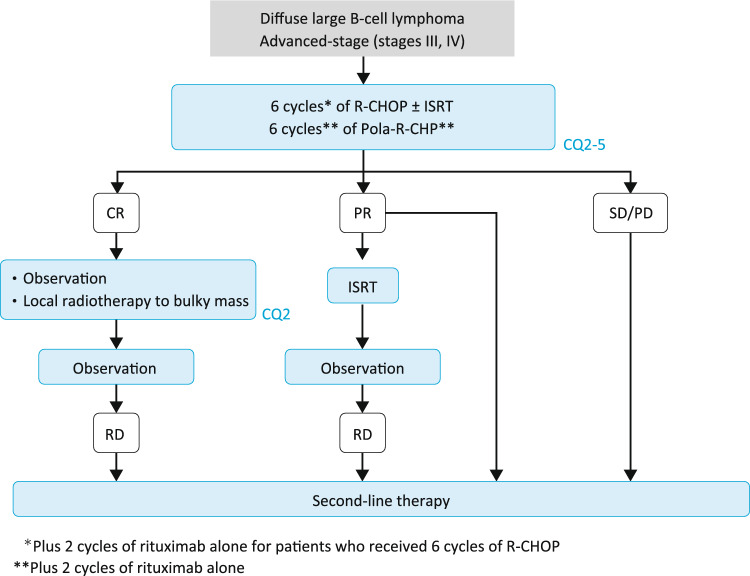
Treatment algorithm for advanced-stage DLBCL

Although 6 to 8 cycles of R-CHOP is the standard chemotherapy for CD20-positive advanced DLBCL [19], 6 cycles of pola-R-CHP (plus 2 cycles of rituximab alone) is another recommended standard therapy option for patients aged 18 to 80 years with an IPI score of 2 or higher (CQ2–5) [15]. The standard number of cycles of R-CHOP is 6 to 8, but no evidence regarding the number of cycles is available due to a lack of prospective clinical trials comparing 6 and 8 cycles. The number of cycles is determined with consideration to eligibility for chemotherapy, based on factors such as age and comorbidities (Fig. 2).

Observation should be selected for patients who show a CR on R-CHOP [20]. Despite the lack of clear evidence that addition of consolidation radiotherapy after chemotherapy improves survival [21], it is reasonable to consider ISRT after chemotherapy because additional irradiation of the former disease site may improve prognosis in elderly patients with a bulky mass (≥ 7.5 cm) before treatment [16]. Although consolidation by high-dose chemotherapy with autologous hematopoietic stem cell transplantation (HSCT) may improve prognosis in younger patients classified in the higher IPI risk groups [22], there is insufficient evidence to recommend this approach in routine practice, and it should only be performed as part of a properly planned clinical trial (CQ2).

If the response is PR or worse, residual disease is confined to one target volume, and salvage therapy is not feasible, local radiotherapy should be performed.

### Algorithm 3

**Fig. 3:**
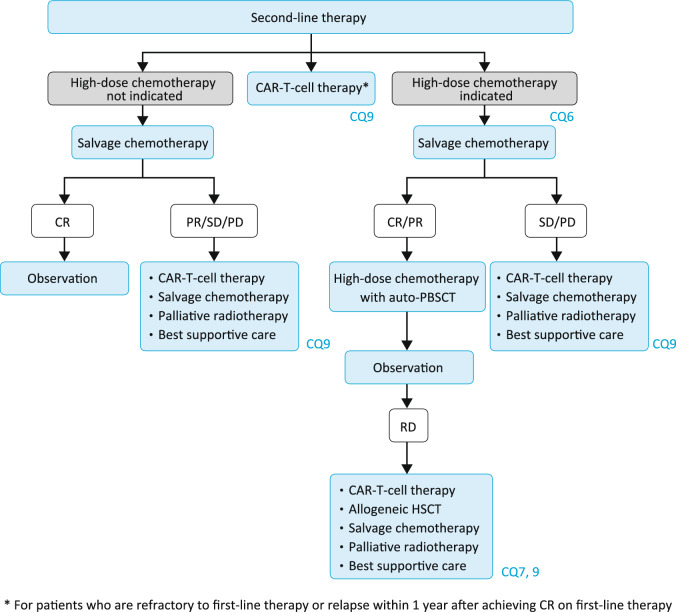
Treatment algorithm for relapsed or refractory DLBCL

For relapsed or refractory DLBCL, high-dose chemotherapy with autologous HSCT is recommended as the standard therapy in responders (CR or PR) to salvage therapy who are judged to be eligible based on age and other relevant factors [23]. Chimeric antigen receptor T-cell (CAR-T-cell) therapy with axicabtagene ciloleucel (axi-cel) or lisocabtagene maraleucel (liso-cel) is an option for patients who are refractory to first-line therapy or relapse within 1 year after achieving CR on first-line therapy, and CAR-T-cell therapy with axi-cel, liso-cel, or tisagenlecleucel (tisa-cel) is an option for patients who are ineligible for or relapse after high-dose chemotherapy with autologous HSCT, including those who do not respond to salvage therapy (CQ9) (Fig. 3) [24, 25].

As the relative benefit of the various salvage chemotherapy options for relapsed or refractory DLBCL is unclear, any of the below-listed regimens can be selected.

Salvage chemotherapy regimens

**Table:** 

DHAP (dexamethasone, cisplatin, and cytarabine) (+ rituximab [R]) [26, 27]
(R-)ESHAP (methylprednisolone, etoposide, cytarabine, and cisplatin) [28]
(R-)ICE (ifosfamide, carboplatin, and etoposide) [29]
CHASE(R) (cyclophosphamide, cytarabine, dexamethasone, and etoposide) [30]
Dose-adjusted (DA)-EPOCH(-R) (etoposide, prednisolone, vincristine, cyclophosphamide, and doxorubicin) [31]
MINE regimen (mitoxantrone, ifosfamide, mesna, and etoposide) [32]
GDP (gemcitabine, dexamethasone, and cisplatin) [33]
BR (bendamustine and rituximab)* [34]
BR plus polatuzumab vedotin* [35]

*When high-dose chemotherapy with autologous HSCT is not indicated

Allogeneic HSCT is a conceivable option for patients relapsed on or refractory to high-dose chemotherapy with autologous HSCT, but is recommended to only be performed in a clinical trial due to its high treatment-related mortality (TRM) and insufficient evidence to support its clinical benefit (CQ7).

## CQ1 What treatments are recommended for newly diagnosed limited-stage DLBCL?

**Table:** 

Recommendation grade: Category 1
Six cycles of R-CHOP performed at a 3-week interval is recommended. Another recommended standard therapy for patients aged 18 to 80 years with an IPI score of 2 or higher is 6 cycles of pola-R-CHP
Recommendation grade: Category 2A
CMT consisting of 3 cycles of R-CHOP performed at a 3-week interval, followed by ISRT, is recommended
Recommendation grade: Category 1
Another recommended standard therapy for patients aged 60 years or younger with an age-adjusted IPI score of 0 is 4 cycles of R-CHOP plus 2 cycles of rituximab

### Explanation

In this section, limited-stage DLBCL corresponds to clinical stage I and contiguous stage II in the Ann Arbor classification, because when considering radiotherapy for limited-stage disease, IFRT was considered to be indicated for contiguous stage II disease treatable within a single radiation field. In current practice, ISRT is used to further reduce the irradiated volume.

The Southwest Oncology Group (SWOG) S8736 trial, which was conducted before the introduction of rituximab, compared 8 cycles of CHOP against CMT consisting of 3 cycles of CHOP followed by IFRT and found that CMT yielded superior progression-free survival (PFS) and overall survival (OS) in the short term, but not in the long term [11, 36]. In the 2000s, the MabThera International Trial (MInT), in which 72% of patients had limited-stage (clinical stage I/II) disease, showed that 6 cycles of chemotherapy with rituximab yielded superior event-free survival (EFS) and OS compared with chemotherapy without rituximab [13], and led to the recognition of 3 cycles of R-CHOP plus IFRT or 6 to 8 cycles of R-CHOP as the standard of care for limited-stage DLBCL. Although outcomes of 6 versus 8 cycles of R-CHOP for limited-stage DLBCL have never been reported, recent clinical trials have used 6 cycles of R-CHOP, and a sub-analysis of the GOYA trial, in which the majority of patients had advanced disease, showed that 6 cycles of R-CHOP may be sufficient [37].

A rituximab-era phase II SWOG (S0016) trial of CMT in patients with limited-stage DLBCL with at least one prognostic factor in the stage-modified IPI (which considers older than 60 years, stage II disease and high LDH to be prognostic factors) showed that combination of 3 cycles of R-CHOP with radiotherapy (40 to 46 Gy) yielded favorable treatment outcomes [12]. A randomized non-inferiority trial by the Lymphoma Study Association (LYSA) was conducted in patients with limited-stage DLBCL without a bulky mass (> 7 cm) who showed a CR on interim PET after 4 cycles of R-CHOP-14. This trial compared outcomes between treatments with and without addition of IFRT after the 4 cycles of R-CHOP-14 in patients who had no prognostic factors in the stage-modified IPI and after an additional 2 cycles of R-CHOP-14 in patients who had one or more prognostic factors. The trial showed that chemotherapy alone was non-inferior to CMT with respect to EFS and OS in patients who showed a CR after 4 cycles of R-CHOP-14 [38]. In another phase II SWOG (S1001) trial conducted in patients with limited-stage DLBCL without a bulky mass (> 10 cm), patients received 3 cycles of R-CHOP, after which point patients with a negative PET scan received 1 additional cycle of R-CHOP, whereas those with a positive PET scan received IFRT and ibritumomab tiuxetan. The 5-year PFS rate was favorable in both the negative PET (89%) and positive PET (86%) groups [39].

A randomized non-inferiority trial by the German Lymphoma Alliance that compared 6 cycles of R-CHOP against 4 cycles of R-CHOP plus 2 cycles of rituximab in patients with limited-stage DLBCL without a bulky mass (> 7.5 cm) and without age-adjusted IPI risk factors showed that 4 cycles of R-CHOP plus 2 cycles of rituximab was non-inferior to 6 cycles of R-CHOP, with a 3-year PFS rate of 96% versus 94% [40]. Early rituximab intensification has not improved outcomes [41, 42].

The POLARIX trial compared 6 cycles of R-CHOP (plus 2 cycles of rituximab alone) against 6 cycles of pola-R-CHP (plus 2 cycles of rituximab alone) in patients aged 18 to 80 years with an IPI score of 2 or higher, 11% of whom had clinical stage I or II disease. Pola-R-CHP yielded a significantly superior primary endpoint of PFS, with a hazard ratio of 0.73 (95% CI 0.57–0.95, *p* = 0.02) [15].

On the basis of this evidence, the recommended therapy for limited-stage DLBCL is either CMT with 3 cycles of R-CHOP plus ISRT or 6 cycles of R-CHOP without ISRT. However, R-CHOP alone is currently the more common choice in daily practice. Another recommended standard therapy option for patients aged 18 to 80 years with an IPI score of 2 or higher is 6 cycles of pola-R-CHP. Four cycles of R-CHOP plus 2 cycles of rituximab is another option for patients without age-adjusted IPI risk factors. Four cycles of R-CHOP may be sufficient for patients with a negative FDG-PET/CT scan after 3 or 4 cycles of R-CHOP.

## CQ2 What treatments are recommended for newly diagnosed advanced DLBCL?

**Table:** 

Recommendation grade: Category 1
Although 6 to 8 cycles of R-CHOP performed at a 3-week interval is the recommended standard therapy, 6 cycles of pola-R-CHP is another standard therapy option recommended for patients aged 18 to 80 years with an IPI score of 2 or higher

### Explanation

Results of several clinical trials from the early 2000s that compared CHOP against R-CHOP established R-CHOP as the standard of care for advanced DLBCL. Since then, various treatments have been investigated for their potential to surpass R-CHOP. Several groups have investigated high-dose chemotherapy with autologous HSCT in first remission, but no controlled trial has shown an OS benefit from this approach, and a meta-analysis of these trials showed no PFS or OS benefit [43]. A trial conducted by the American group Alliance that compared R-CHOP with DA-EPOCH-R (etoposide, prednisolone, vincristine, cyclophosphamide, doxorubicin, and rituximab) showed no difference in PFS and OS between regimens, and higher toxicity with DA-EPOCH-R [44]. In addition, large controlled trials conducted inside and outside Japan have investigated other recently introduced drugs that inhibit molecules in the signaling pathway in combination with R-CHOP [4–6] or in maintenance therapy after remission [20, 45], as well as replacement of rituximab with obinutuzumab [46], but none of these treatments yielded better survival than R-CHOP.

A trial reported in 2021 that compared 6 cycles of R-CHOP (plus 2 cycles of rituximab alone) against 6 cycles of pola-R-CHP (plus 2 cycles of rituximab alone) in patients aged 18 to 80 years with an IPI score of 2 or higher showed that pola-R-CHP yielded a significantly superior 2-year PFS rate compared with R-CHOP (76.7% vs. 70.2%). Although OS did not differ between regimens, pola-R-CHP had a 25% lower incidence of events such as progression, relapse, death, and transition to next line therapy [15], and is, therefore, recommended as a standard treatment for abovementioned patients. However, the safety and efficacy of pola-R-CHP have only been established in this particular patient group, and its superiority over R-CHOP in other patients is unknown.

The standard number of cycles of R-CHOP is 6 to 8, but no evidence regarding the number of cycles exists due to a lack of prospective clinical trials comparing 6 and 8 cycles. In a subanalysis of a trial that compared R-CHOP with obinutuzumab plus CHOP, 3-year PFS rates did not differ between patients who received 6 versus 8 cycles of R-CHOP [15, 37].

On the basis of the above evidence, although 6 to 8 cycles of R-CHOP performed at a 3-week interval is the recommended standard therapy for newly diagnosed advanced DLBCL, 6 cycles of pola-R-CHP (plus 2 cycles of rituximab alone) is another standard therapy option recommended for patients aged 18 to 80 years with an IPI score of 2 or higher.

## CQ3 What treatments are recommended to prevent central nervous system relapse in DLBCL?

**Table:** 

Recommendation grade: Category 2A
Addition of intrathecal chemotherapy to R-CHOP is recommended for prevention of central nervous system (CNS) relapse in primary testicular DLBCL
Recommendation grade: Category 2B
Addition of intrathecal chemotherapy or high-dose methotrexate may reduce the incidence of CNS relapse in patients judged to be high risk for CNS relapse according to a predictive model, patients with involvement of specific extranodal sites, and CD5-positive patients, but the benefit of these treatments has not been established

### Explanation

The incidence of CNS relapse in DLBCL has been reported as 2.8 to 7.1% [47–49]. Due to this low incidence, routine CNS prophylaxis is not recommended for all patients, and the conventional approach has been to limit CNS prophylaxis to patients at high risk of CNS relapse. Although chemotherapy regimens with added rituximab may reduce the risk of CNS relapse [49], CNS prophylaxis may be indicated for high-risk patients with a CNS-IPI (a predictive model for CNS relapse that composed of five factors of the IPI plus renal/adrenal involvement) score of 4 or higher [48], patients with involvement of extranodal sites such as the testes, bone marrow, paranasal sinuses/nasal cavity, orbit, bone, peripheral blood, breast, kidneys/adrenal glands, uterus, ovaries, or fallopian tubes, and CD5-positive patients [50]. A large retrospective analysis of primary testicular DLBCL by the International Extranodal Lymphoma Study Group showed that 52% of 373 patients relapsed and the 10-year cumulative incidence of CNS relapse was high (15%) [51]. The same group conducted a prospective phase II trial of 6 to 8 cycles of R-CHOP and 4 intrathecal injections of methotrexate plus contralateral testis irradiation (and irradiation of sites of nodal involvement in patients with stage II disease) in patients with newly diagnosed stage I or II primary testicular DLBCL and found that the 5-year cumulative incidence of CNS relapse was 6% [52]. These results are why prophylactic intrathecal chemotherapy and irradiation are recommended for primary testicular DLBCL. Outcomes for Burkitt lymphoma and adult T-cell leukemia/lymphoma have led to widespread use of intrathecal chemotherapy for prevention of CNS relapse. A systematic review of 7357 DLBCL patients treated with chemotherapy regimens with added rituximab or obinutuzumab and a retrospective analysis of 989 DLBCL patients treated with R-CHOP showed that prophylactic intrathecal chemotherapy may be insufficient to prevent CNS relapse [53, 54]. Another option for CNS prophylaxis is high-dose methotrexate. In a study of 65 patients with DLBCL at high risk of CNS relapse who received at least one cycle of high-dose methotrexate in addition to R-CHOP, the 3-year cumulative CNS relapse rate was only 3% [55]. In a phase II trial in which 47 patients with previously untreated CD5-positive DLBCL received 4 cycles of DA-EPOCH-R (etoposide, prednisolone, vincristine, cyclophosphamide, doxorubicin, and rituximab) followed by 2 cycles of high-dose methotrexate and an additional 4 cycles of DA-EPOCH-R, the 2-year CNS relapse rate was 9% [56]. Although these results suggest a potential benefit of high-dose methotrexate, other studies have shown conflicting results, and thus no preventive therapy has been established [57].

## CQ4 What chemotherapy regimens are appropriate for patients with newly diagnosed DLBCL who presumably have reduced cardiac function?

**Table:** 

Recommendation grade: Category 2A
Administration of a reduced dose of doxorubicin, discontinuation of doxorubicin, or switching to continuous infusion of doxorubicin should be considered. The same regimens with doxorubicin switched to another drug may be options for alternative therapies, but the clinical benefit of such regimens has not been verified

### Explanation

One of the major toxic effects of doxorubicin is myocardial damage. The risk of heart failure due to myocardial damage is correlated with the cumulative dose of doxorubicin: incidence is only approximately 0.14% at a total dose less than 400 mg/m^2^, but increases to 7% at 550 mg/m^2^ and 18% when the dose exceeds 700 mg/m^2^ [58]. A meta-analysis of 49,017 cancer patients who received doxorubicin-containing chemotherapy showed that the rate of symptomatic cardiotoxicity over a median observation period of 9 years was 6% [59]. A meta-analysis of R-CHOP/CHOP showed that the incidence of heart failure was 2% [60]. A large retrospective study of elderly patients (≥ 65 years) with DLBCL who received doxorubicin-containing chemotherapy showed that the risk of heart failure did not increase in patients who received 6 or fewer cycles of chemotherapy, and that only hypertension was a risk factor for heart failure [61]. These findings reflect the fact that a large percentage of elderly patients with DLBCL have reduced cardiac function and other risk factors for heart failure such as hypertension, dyslipidemia, and diabetes present even before treatment. A retrospective analysis from the United States in patients with intermediate-grade NHL who received CHOP (cyclophosphamide, doxorubicin, vincristine, and prednisolone) compared patients who discontinued treatment early (before 6 cycles) with those who completed at least 6 cycles and showed that early discontinuation was associated with poor survival in patients younger than 75 years but not in those aged 75 years and older [62]. Another large cohort study from the United States that compared DLBCL patients aged 80 years and older who received either at least 85% or less than 85% of the doxorubicin dose in the first cycle showed that TRM was higher and a 1-year survival rate was significantly poorer among those who received at least 85% of the dose [63]. In chemotherapy for DLBCL, although the actual relative dose intensity of doxorubicin is correlated with prognosis [64], dose reduction is recommended based on the findings described above to reduce the risk of doxorubicin-induced myocardial damage in elderly adults who presumably have reduced cardiac function. No clinically significant cardiotoxicity was observed in a phase II trial of DA-EPOCH (etoposide, prednisolone, vincristine, cyclophosphamide, and doxorubicin) for relapsed or refractory NHL [65], which suggests that switching the route of doxorubicin administration from bolus injection to continuous infusion may reduce cardiotoxicity. Many trials have investigated R-COMP (rituximab, cyclophosphamide, non-pegylated liposomal doxorubicin [NPLD], vincristine, and prednisolone), which substitutes NPLD for doxorubicin [66–69], and meta-analyses have shown that R-COMP is non-inferior to R-CHOP with respect to response rate, OS, and PFS [70]. Studies that investigated treatment options for DLBCL patients with reduced cardiac function were a phase II trial of R-GCVP (rituximab, gemcitabine, cyclophosphamide, vincristine, and prednisolone), which substitutes gemcitabine for doxorubicin [71], a phase III trial of R-CEOP (rituximab, cyclophosphamide, epirubicin, vincristine, and prednisolone), which substitutes epirubicin for doxorubicin [72, 73], and a retrospective study of R-CEOP (rituximab, cyclophosphamide, etoposide, vincristine, and prednisolone), which substitutes etoposide for doxorubicin [74]. All of these regimens showed relatively good tolerability and survival, which makes them promising candidates for alternative treatments. A randomized phase III trial of R-CHOP versus R-THP-COP (rituximab, cyclophosphamide, pirarubicin, vincristine, and prednisolone), which substitutes pirarubicin for doxorubicin [75], and a meta-analysis of CNOP (cyclophosphamide, mitoxantrone, vincristine, and prednisolone), which substitutes mitoxantrone for doxorubicin [76], showed no difference in cardiotoxicity compared with CHOP, and thus these results do not constitute evidence for alternative treatments.

## CQ5 What is the standard therapy for DLBCL in elderly patients?

**Table:** 

Recommendation grade: Category 1
Patients younger than 80 years are treated the same as younger patients: limited-stage disease is treated with either CMT consisting of 3 cycles of R-CHOP followed by radiotherapy or 6 to 8 cycles of R-CHOP alone, and advanced disease is treated with 6 to 8 cycles of R-CHOP. Standard-dose R-CHOP is recommended. Another recommended standard therapy for patients aged 18 to 80 years with an IPI score of 2 or higher is 6 cycles of pola-R-CHP
Recommendation grade: Category 2A
Reduced-dose or reduced-cycle R-CHOP are suitable options for patients 80 years and older

### Explanation

In the WHO classification, “elderly” is defined as age 65 years or older. Age of 61 years or older is an unfavorable prognostic factor in the IPI, meaning that old age itself is associated with a poor prognosis. Elderly patients have reduced organ function, comorbidities and complicating conditions, cognitive impairment, and social frailty. They are also known to have higher rates of TRM and toxicity than younger patients, and thus have generally been treated with reduced-dose or reduced-cycle R-CHOP [77]. Most clinical trials of DLBCL set an upper age limit of 80 years, and R-CHOP is the standard therapy in patients up to 80 years old who have generally good performance status and organ function and no major comorbidities [41, 78]. Another recommended standard therapy for patients aged 18 to 80 years with an IPI score of 2 or higher is 6 cycles of pola-R-CHP [15].

A phase II Groupe d’Etude des Lymphomes de l’Adulte (GELA) trial that investigated 6 cycles of low-dose R-CHOP in very elderly patients (≥ 80 years) with newly diagnosed disease of all stages showed a low incidence of toxicity requiring inpatient treatment, which demonstrated that curative treatment is still possible in patients older than 80 years [79]. Consequently, the dose intensity of R-CHOP must be maintained in patients younger than 80 years who have adequate performance status and organ function. An optimal strategy of treatment for patients 80 years and older has not been established due to the difficulty of conducting large clinical trials in that population. The results of the phase II trial [15] and meta-analysis [80, 81] described above indicate that reduced-dose R-CHOP is preferable. Comprehensive geriatric assessment tools have been shown to be useful for pretreatment evaluation to objectively identify patients who are ineligible for standard therapy [82–84].

Regimens shown to be non-inferior to R-CHOP in phase III trials include R-THP-COP (rituximab, cyclophosphamide, pirarubicin, vincristine, and prednisolone) [75], which substitutes pirarubicin for doxorubicin, and R-CEOP (rituximab, epirubicin, cyclophosphamide, vincristine, and prednisolone) [73], which substitutes epirubicin for doxorubicin. A prospective registry study of reduced-dose EPOCH-R (etoposide, prednisolone, vincristine, cyclophosphamide, doxorubicin, and rituximab) [85] in very elderly patients (≥ 75 years) showed 3-year OS and PFS rates of 62.8% and 60.8%, respectively. These three regimens are options when reduced-dose R-CHOP is not feasible. R-COMP (rituximab, cyclophosphamide, NPLD, vincristine, and prednisolone), which substitutes liposomal doxorubicin for doxorubicin, showed comparable efficacy to R-CHOP in a meta-analysis [70] and a phase II trial [69]. In addition, single-arm phase II trials showed that BR (bendamustine and rituximab) [69] had a 2-year OS rate of 51% [86], and R-GemOx (rituximab, gemcitabine, and oxaliplatin) [86] had a 3-year OS rate of 65% [87], with both regimens showing acceptable toxicity. Japanese National Health Insurance does not cover bendamustine for newly diagnosed patients or liposomal doxorubicin or oxaliplatin for malignant lymphoma.

Standard therapy for elderly patients with relapsed or refractory DLBCL has not been established due to a lack of large clinical trials. A phase I/IIb trial of BR plus polatuzumab vedotin [35], retrospective analyses of R-GCVP (rituximab, gemcitabine, cyclophosphamide, vincristine, and prednisolone) [88] and fractionated ICE (ifosfamide, carboplatin, and etoposide) [89], and a sub-analysis of a phase I/II trial of cellular therapy [90] showed that these regimens may be treatment options. However, the appropriate salvage therapy must be selected with consideration to the patient’s performance status and organ function as well as the properties of each salvage therapy.

## CQ6 Is high-dose chemotherapy with autologous hematopoietic stem cell transplantation recommended for relapsed or refractory DLBCL?

**Table:** 

Recommendation grade: Category 1
High-dose chemotherapy with autologous HSCT is recommended for patients who respond to salvage therapy (CR or PR) and meet age criteria and other eligibility criteria
Recommendation grade: Category 1
Axi-cel or liso-cel may offer a better prognosis than high-dose chemotherapy with autologous HSCT for patients who are refractory to first-line therapy or relapse within 1 year of achieving CR on first-line therapy

### Explanation

As relapsed or refractory DLBCL has a poor long-term prognosis with conventional salvage therapy alone, studies have investigated whether salvage therapy followed by consolidation high-dose chemotherapy with autologous HSCT could improve prognosis. One controlled trial (PARMA trial) demonstrated the superiority of high-dose chemotherapy with autologous HSCT for relapsed or refractory intermediate- to high-grade lymphomas, most of which were DLBCL [23]. Patients who responded (CR or PR) after 2 cycles of DHAP (dexamethasone, cisplatin, and cytarabine) were assigned to receive either 4 additional cycles of DHAP plus radiotherapy or high-dose chemotherapy with autologous HSCT plus radiotherapy, and outcomes were compared. High-dose chemotherapy with autologous HSCT was found to yield better 5-year OS and EFS rates. No controlled trial has evaluated the effect of adding high-dose chemotherapy with autologous HSCT in the rituximab era. But trials that compared salvage therapy for relapsed or refractory DLBCL and intermediate- and high-grade lymphoma, as well as a large study of survival in relapsed or refractory DLBCL, showed that not undergoing high-dose chemotherapy with autologous HSCT resulted in a poorer prognosis, and the survival outcomes of the PARMA trial were replicated in those who underwent high-dose chemotherapy with autologous HSCT [26, 91, 92].

In 2021, results were reported from three clinical trials that compared high-dose chemotherapy with autologous HSCT against CAR-T-cell therapy in patients who were refractory to first-line therapy or relapsed within 1 year after achieving CR on first-line therapy. The results showed that axi-cel and liso-cel were significantly superior to high-dose chemotherapy with autologous HSCT in terms of EFS [24, 25], but tisa-cel was not superior [93]. Although direct comparison of these three trials is difficult due to differences in whether or not bridging therapy was performed and the time from lymphocyte apheresis to CAR-T product infusion, they show that axi-cel or liso-cel may improve prognosis in patients who are refractory to first-line therapy or relapse within 1 year of achieving CR on first-line therapy, such as the patient populations in these trials.

On the basis of the above evidence, axi-cel or liso-cel is recommended for patients who are refractory to first-line therapy or relapse within 1 year of achieving CR on first-line therapy, whereas high-dose chemotherapy with autologous HSCT is recommended for transplant-eligible patients with relapsed or refractory DLBCL who respond (CR or PR) to salvage therapy. However, it should be noted that indications for CAR-T-cell therapy for relapsed or refractory DLBCL are expanding as evidence of its efficacy accumulates.

## CQ7 Is allogeneic hematopoietic stem cell transplantation recommended for relapsed or refractory DLBCL?

**Table:** 

Recommendation grade: Category 2B
Allogeneic HSCT is recommended an option for patients with refractory disease or patients relapsed on high-dose chemotherapy with autologous HSCT

### Explanation

High-dose chemotherapy with autologous HSCT is the first option to consider for relapsed or refractory DLBCL because allogeneic HSCT has a higher rate of TRM and does not yield superior OS [94]. As DLBCL relapsed on or refractory to high-dose chemotherapy with autologous HSCT has a very poor prognosis [92], some kind of treatment as part of a clinical trial is recommended, and allogeneic HSCT is one such option.

There is little data from large prospective trials on allogeneic HSCT for DLBCL, and most studies are retrospective analyses. A large retrospective study using registry data from the Center for International Blood and Marrow Transplant Research and the European Society for Blood and Marrow Transplantation compared outcomes for non-myeloablative allogeneic HSCT using haploidentical, HLA-matched related, and HLA-matched unrelated donor bone marrow, and found that 3-year non-relapse TRM did not differ (22%, 17%, 26–30%) and 3-year OS rates were comparable (46%, 50%, 43–46%) between groups [95]. In a Japanese multicenter retrospective study of allogeneic HSCT for relapsed or refractory DLBCL, the 4-year OS rate was 23%, RFS rate was 20%, the cumulative incidence of non-relapse TRM was 23%, and the cumulative incidence of relapse was 57% [96]. Particularly good 4-year OS and RFS rates (46% and 36%, respectively) were reported for patients who showed a good response at the time of transplantation. These results suggest that some patients may achieve long-term survival with allogeneic HSCT.

In summary, allogeneic HSCT should be considered as one option for patients relapsed on or refractory to high-dose chemotherapy with autologous HSCT.

## CQ8 What DLBCL features or entities (e.g., extranodal lymphoma) require special treatment considerations?

**Table:** 

Recommendation grade: Category 2B
Subtype-specific therapy may be effective for primary CNS DLBCL, primary testicular DLBCL, and primary mediastinal large B-cell lymphoma

### Explanation

Special treatment considerations may be effective for some subtypes and related entities of DLBCL as defined in the 2017 WHO classification, as well as for other extranodal DLBCL with characteristic pathological features or DLBCL with characteristic biomarkers.

When treating primary CNS DLBCL, it has conventionally been recommended to first perform high-dose methotrexate-based chemotherapy before cranial irradiation to ensure drug delivery to the CNS [97]. However, cranial irradiation has a high incidence of delayed CNS damage, so thiotepa-based high-dose chemotherapy with autologous HSCT is being investigated as an alternative. In a phase II trial conducted at Memorial Sloan Kettering Cancer Center, patients with CR after 5 cycles of R-MPV (rituximab, methotrexate, procarbazine, and vincristine) received high-dose chemotherapy with autologous HSCT. Patients with PR or SD received 2 additional cycles of R-MPV, and those who responded (PR or CR) to the additional cycles received high-dose chemotherapy with autologous HSCT. With this approach, the 2-year PFS and OS rates were 79% and 81%, respectively [98]. In a randomized phase II trial conducted by the International Extranodal Lymphoma Study Group (IELSG) (IELSG32 trial), patients who achieved SD or better on chemotherapy underwent autologous peripheral blood stem cell collection followed by cranial irradiation or high-dose chemotherapy with autologous HSCT, and the 2-year PFS rate was 80% with cranial irradiation versus 69% with high-dose chemotherapy with autologous HSCT [99]. Therefore, high-dose chemotherapy with autologous HSCT is an alternative option to cranial irradiation. Bruton’s tyrosine kinase inhibitors have also demonstrated efficacy in relapsed or refractory primary CNS DLBCL [100].

For limited-stage primary testicular DLBCL, good survival rates have been reported with R-CHOP plus intrathecal chemotherapy and contralateral testis irradiation, and thus this is the recommended approach [51, 52]. For primary mediastinal large B-cell lymphoma, a phase II trial of DA-EPOCH-R (etoposide, prednisolone, vincristine, cyclophosphamide, doxorubicin, and rituximab) reported a 5-year EFS rate of 93%, indicating that radiotherapy after chemotherapy can be omitted and that DA-EPOCH-R is efficacious [101]. However, no trials have compared DA-EPOCH-R against R-CHOP plus radiotherapy, and thus the necessity of radiotherapy for patients who are FDG-PET/CT-negative after R-CHOP remains inconclusive at this time. For intravascular large B-cell lymphoma (IVLBCL) with no obvious CNS involvement at diagnosis, a phase II trial demonstrated the efficacy of R-CHOP plus high-dose methotrexate and intrathecal chemotherapy [102]. In previously untreated clinical stage II to IV CD5-positive DLBCL, a phase II trial demonstrated the efficacy of DA-EPOCH-R plus high-dose methotrexate [56]. As both IVLBCL and CD5-positive DLBCL are rare, the these treatment approaches are options.

It is recommended to follow the DLBCL guidelines for other subtypes of extranodal DLBCL and subtypes listed as classified under DLBCL in the Overview section.

## CQ9 Is CAR-T-cell therapy recommended for relapsed or refractory DLBCL?

**Table:** 

Recommendation grade: Category 1
CAR-T-cell therapy is one recommended treatment for relapsed or refractory DLBCL that failed to achieve a CR with first-line therapy or that relapsed within 12 months. However, it is recommended to make an appropriate decision regarding the indication

### Explanation

In Japan, three CAR-T products (tisa-cel, axi-cel, and liso-cel) are used for the treatment of malignant lymphoma, and all of these products target the CD19 antigen. They are approved for relapsed or refractory DLBCL not previously treated with an anti-CD19 CAR-T product, but the requirements for approval vary between products. The three products differ in whether CD28 or 4-1BB is used as a co-stimulatory molecule, but the difference in efficacy among the three products is unclear [103].

CR rates reported in phase II trials of tisa-cel, axi-cel, and liso-cel for relapsed or refractory DLBCL (JULIET, ZUMA-1, and TRANSCEND NHL 001, respectively) range from 40 to 54% [104–106]. In long-term results of the JULIET trial, the CR rate was 39% at a median follow-up of 40.3 months, and median PFS was not reached in patients who achieved CR [107]. Other studies have also shown a durable response in patients who achieve CR [108, 109]. In actual clinical practice, a certain response rate has also been observed in patients more heavily pretreated than clinical trial participants and in patients ineligible for participation in clinical trials [110, 111]. CAR-T products have also been compared against conventional therapies. Studies that used propensity analysis or matching-adjusted indirect comparison to compare outcomes of the SCHOLAR-1, a large retrospective study of chemotherapy in relapsed or refractory DLBCL, against outcomes of the ZUMA-1 and TRANSCEND NHL 001 trials, showed that CAR-T-cell therapy may offer better efficacy than conventional therapies in terms of response rate and survival [112, 113].

In addition, in a phase III trial (ZUMA-7) that evaluated the efficacy of axi-cel as second-line therapy for relapsed or refractory aggressive lymphoma or large B-cell lymphoma, standard high-dose chemotherapy with autologous HSCT was compared against CAR-T-cell therapy in patients with relapsed or refractory DLBCL that failed to achieve a CR with first-line therapy or that relapsed within 12 months but responded to platinum-based salvage chemotherapy. At a median follow-up of 24.9 months, median EFS was 8.3 months with CAR-T-cell therapy versus 2.0 months with standard therapy, and the 2-year EFS rate was significantly better with CAR-T-cell therapy (41% vs. 16%) [24]. Similar trials were conducted for tisa-cel (BELINDA) and liso-cel (TRANSFORM). In the TRANSFORM trial, CAR-T-cell therapy yielded better EFS than standard therapy, but in the BELINDA trial, CAR-T-cell therapy did not yield better EFS than standard therapy [25, 93]. The results of these three trials led to the approval of axi-cel and liso-cel for second-line therapy. Direct comparison of these three trials is difficult due to differences in whether or not bridging therapy was performed and the time from lymphocyte apheresis to CAR-T product infusion.

Although CAR-T-cell therapy requires careful safety management due to characteristic adverse events including cytokine release syndrome and neurotoxicity that sometimes require intensive care [114], it has also been shown to improve health-related quality of life [115].

On the basis of the above evidence, CAR-T-cell therapy is considered efficacious for relapsed or refractory DLBCL, and is one recommended treatment for relapsed or refractory DLBCL that failed to achieve a CR with first-line therapy or that relapsed within 12 months. CAR-T-cell therapy should also be considered as an option for patients with relapsed or refractory DLBCL who are ineligible for high-dose chemotherapy with autologous HSCT including those who relapse on high-dose chemotherapy with autologous HSCT or do not respond to salvage chemotherapy. However, it is recommended to make an appropriate decision regarding the indication for CAR-T-cell therapy, with consideration to the requirements for approval.
